# 2371 Validation of a novel PD-L1 assay for bladder cancer circulating tumor cells

**DOI:** 10.1017/cts.2018.150

**Published:** 2018-11-21

**Authors:** Nicolas Seranio, Louise Aguarin, Jay F. Dorsey, John P. Christodouleas, Gary D. Kao

**Affiliations:** University of Pennsylvania School of Medicine

## Abstract

OBJECTIVES/SPECIFIC AIMS: Bladder cancer patients being considered for immune checkpoint blockade are often judged on immunohistochemical staining for the checkpoint target protein PD-L1 in the original surgery or biopsy sample. However, sampling error or the clinical evolution of most patients’ cancer can render the original PD-L1 assessment no longer accurate. In contrast, circulating tumor cells (CTCs) allow serial noninvasive sampling of the current tumor status throughout a patient’s clinical course including those with the highest metastatic potential. We therefore sought to develop a method for quantifying PD-L1 expression in CTCs towards addressing inherent limitations of current UC management. METHODS/STUDY POPULATION: This work utilizes both cancer cell lines as well as patient samples. Positive and negative control cancer cell lines were assessed via “industry standard” antibodies for PD-L1 expression via Western blots and immunofluorescence, and a threshold-based method was developed for reliable quantification. PDL-1 expression was additionally verified via interferon-mediated up-regulation. CTCs isolated from bladder cancer patient samples via a density centrifugation method were then assessed for PD-L1 via the same antibodies. RESULTS/ANTICIPATED RESULTS: We will show preliminary preclinical and clinical data that validates the sensitivity and specificity of our assay. A case study will be presented that illustrate the potential useful of the novel approach we describe and which should be complementary to current clinical practices. In a patient with metastatic bladder cancer, this method effectively detected the PD-L1 expression in CTCs taken at a time coincident to when the patient derived an excellent response to the PD-L1 checkpoint inhibitor Pembrolizumab. DISCUSSION/SIGNIFICANCE OF IMPACT: This work highlights the potential utility of CTCs in the management of bladder cancer. It may be the case that this assay in conjunction with current methods of patient selection for immunotherapy may allow for better response prediction than either method alone.

